# LncRNA LINC00511 promotes COL1A1-mediated proliferation and metastasis by sponging miR-126-5p/miR-218-5p in lung adenocarcinoma

**DOI:** 10.1186/s12890-022-02070-3

**Published:** 2022-07-16

**Authors:** Yudong Wang, Xingke Mei, Weikang Song, Chen Wang, Xueshan Qiu

**Affiliations:** 1 Thoracic Surgery Department, Shengjing Hospital of China Medical University, Shenyang, 110136 Liaoning China; 2 College of Basic Medical Science, China Medical University, No. 77 Puhe Road, Shenbei New District, Shenyang, 110001 Liaoning China; 3 Department of Pathology, The First Hospital of China Medical University, No. 155 Nanjing North Street, Heping District, Shenyang, 110122 Liaoning China

**Keywords:** Linc00511, Lung adenocarcinoma, miR-126-5p, miR-218-5p, COL1A1, Proliferation/metastasis

## Abstract

**Background:**

Lung adenocarcinoma (LUAD) is currently the leading cause of cancer-related death worldwide. Long noncoding RNAs (lncRNAs) play key roles in tumor occurrence and development as crucial cancer regulators. The present study aimed to explore the molecular mechanism and regulatory network of Linc00511 in LUAD and to identify new potential therapeutic targets for LUAD.

**Methods:**

Real-time quantitative polymerase chain reaction (RT–qPCR) was performed to determine the relative Linc00511 levels in LUAD tissues and cells. The proliferation, apoptosis, migration, and invasion abilities of LUAD cells were assessed by a Cell Counting Kit-8 (CCK-8) assay, a colony formation assay, flow cytometry, and a Transwell assay. Changes in hsa_miR-126-5p, hsa_miR-218-5p, and COL1A1 expression were analyzed using western blotting and RT–qPCR. Targeted binding between miR-126-5p/miR-218-5p and Linc00511 or COL1A1 was verified with a luciferase reporter system and confirmed by an RNA pulldown assay. The participation of the PI3K/AKT signaling pathway was confirmed via western blotting. Xenograft animal experiments were performed to detect the impact of Linc00511 on LUAD tumor growth in vivo.

**Results:**

In the present work, we observed that Linc00511 was upregulated in LUAD tissues and cells. Loss/gain-of-function experiments indicated that knockdown of Linc00511 significantly inhibited LUAD cell proliferation, migration and invasion and promoted LUAD cell apoptosis, whereas overexpression of Linc00511 showed the opposite effects. In addition, we determined that Linc00511 promoted COL1A1-mediated cell proliferation and cell motility by sponging miR-126-5p and miR-218-5p. Moreover, Linc00511 activated the PI3K/AKT signaling pathway through upregulation of COL1A1. Finally, silencing of Linc00511 inhibited LUAD tumor growth in vivo.

**Conclusions:**

Linc00511 acts as a competing endogenous RNA to regulate COL1A1 by targeting miR-126-5p and miR-218-5p, thereby promoting the proliferation and invasion of LUAD cells.

**Supplementary Information:**

The online version contains supplementary material available at 10.1186/s12890-022-02070-3.

## Introduction

As a highly malignant tumor, lung cancer is a serious threat to people’s health and life [1]. Epidemiological investigations have indicated the increasing prevalence of lung cancer in the twenty-first century, and it has become one of the common causes of cancer-related death worldwide [2]. Lung cancer can be classified as small cell lung cancer and non-small-cell lung cancer (NSCLC) depending on its histopathological features [3]. Among lung cancer, lung adenocarcinomas (LUAD) are the most prevalent histological subtype of NSCLC, accounting for approximately 40% of cases [4]. Despite the current advances in clinical treatment, lung adenocarcinoma remains a refractory disease due to the difficulty of its surgical resection and its profound distant metastasis capacity, poor prognosis, and frequent recurrence [5]. The process of LUAD occurrence and development is extremely complicated, and the mechanisms that induce metastasis remain unclear. Consequently, exploring the underlying mechanisms of tumor progression and metastasis and seeking out new metastasis-related targets in LUAD are urgent and needed.

In recent years, the noncoding regions of the genome have been implicated in playing crucial roles in different types of diseases. Long noncoding RNAs (lncRNAs), a novel group of non-protein-coding transcripts, exceed 200 nucleotides in length [6]. LncRNAs, usually by epigenetic, transcriptional, and posttranscriptional mechanisms, play critical roles in multiple biological processes related to tumor cell growth, apoptosis, invasion, and metastasis [7]. Furthermore, as key regulators of tumorigenesis and cancer progression, many kinds of lncRNAs have been observed to be related to the progression of LUAD. For instance, LINC00673-v4 promotes LUAD invasiveness by activating WNT/β-catenin signaling [8]. LINC00472 regulates cellular mechanical properties and inhibits LUAD cell migration and invasion by binding to YBX1 [9]. The competing endogenous RNA (ceRNA) role is a common role played by lncRNAs and must be considered when elucidating the mechanism of action of lncRNAs. In the present study, we hypothesized that Linc00511 acts as a ceRNA to increase COL1A1 expression by targeting miR-126-5p or miR-218-5p, which in turn drives LUAD cell growth, migration, and invasion.

Linc00511 behaves as an oncogene, affecting tumor volume and tumor metastasis and contributing to poor survival rates. Linc00511 was found to be enriched in gastric cancer [10], breast cancer [11] and NSCLC [12] tissues and cell lines and to mediate cell proliferation, invasion, metastasis, and apoptosis. Linc00511 can act as a “sponge” for miR-185-3p, targeting E2F1 and promoting breast cancer tumorigenesis [11]. In gastric cancer, Linc00511 acts as a ceRNA by adsorbing miR-515-5p to boost gastric cancer cell growth and invasion [10]. Previous studies have indicated that differential Linc00511 expression is notably correlated with LUAD patient prognosis [13]. Although previous studies have revealed the molecular mechanism and biological function of Linc00511 in cancers, its specific role in LUAD is still worth exploring. Therefore, we selected Linc00511 as our research focus and continued to investigate the underlying molecular mechanism of its impact on LUAD.

CeRNAs are often thought of as functional mediators between lncRNAs and mRNAs, interacting with the same microRNAs (miRNAs) and mediating new “languages” for communication [14]. MiRNAs have promising applications in the development of LUAD therapies. MiRNAs can precisely interact with the 3’-untranslated regions of mRNAs to posttranscriptionally regulate gene expression [15]. Some miRNAs can not only take part in tumor cell proliferation and metastasis but also be considered prognostic factors in LUAD [16]. Current works have shown that both miR-126-5p and miR-218-5p play biological roles in cancers through a ceRNA mechanism. For example, the lncRNA PVT1-5 functions as a ceRNA of miR-126-5p to boost lung cancer cell proliferation by activating miR-126-5p/SLC7A5 signaling [17]. However, to date, there is a lack of systematic studies on miR-126-5p and miR-218-5p expression levels in LUAD and their mechanisms of action. Collagen type I alpha 1 chain (COL1A1) is confirmed to be an extracellular matrix protein that is overexpressed in many types of malignant tumors, including lung, breast, and colorectal tumors [18–20]. In addition, differential expression of COL1A1 predicts poor clinical outcomes (e.g., lymph node metastasis) in lung squamous cell carcinoma patients [21]. Thus, COL1A1 is considered a prognostic marker for lung tumors and a potential therapeutic target. It is the basis for the inhibition of cancer cell survival and metastasis. However, the potential molecular mechanism of COL1A1 in LUAD needs to be clarified.

The research presented here, consistent with previous results, showed that Linc00511 expression was greatly increased in LUAD tissues and cell lines. Silencing Linc00511 inhibited LUAD cell proliferation, migration, and invasion. Interestingly, Linc00511 acted as a sponge of miR-126-5p and miR-218-5p, targeting COL1A1 and thus playing an initiating role in LUAD pathogenesis. Further studies on the downstream targets of Linc00511 in LUAD demonstrated that Linc00511 participated in activating the phosphatidylinositol 3-kinase/AKT (PI3K/AKT) signaling pathway in cells to induce the malignant progression of LUAD. The biological role of Linc00511 in LUAD tumor growth was examined in vivo in a xenograft tumor model, and its detailed interactions with miR-126-5p, miR-218-5p, and COL1A1 were further identified. In conclusion, Linc00511 exerts a crucial effect on the initiation and prognosis of LUAD and is a promising diagnostic and prognostic biomarker.

## Materials and methods

### Clinical tissue acquisition

LUAD tissues and adjacent normal tissues were obtained from Shengjing Hospital of China Medical University, and each participant signed an informed consent form. The inclusion criteria were as follows: (1) all patients were pathologically diagnosed with lung adenocarcinoma; (2) no patient received any chemotherapy or radiotherapy before surgery; and (3) all patients had complete clinicopathological data available. Tissues were frozen in liquid nitrogen and stored in a − 80 °C freezer.

### Cell culture

Two LUAD cell lines (A549 and PC9) and one normal bronchial epithelial cell line (BEAS-2B) were obtained from the Cell Bank of the Chinese Academy of Sciences (Kunming, China). All cell lines were cultured in RPMI 1640 medium (Gibco, USA) supplemented with 10% fetal bovine serum (FBS; Invitrogen) and 1% penicillin/streptomycin (Gibco, USA) in an atmosphere of 5% CO_2_ at 37 °C.

### Cell transfection

Linc00511 small interfering RNAs (si-linc00511 #1 and si-linc00511 #2) and the corresponding siRNA control (si-nc), miR-126-5p or miR-218-5p mimics/inhibitors and their corresponding controls (miR-NCs) were synthesized by GenePharma (Shanghai, China). The Linc00511 and COL1A1 sequences were inserted into the pcDNA3.1 vector (Invitrogen) to overexpress Linc00511 and COL1A1 (pcDNA3.1-linc00511, oe-linc00511; pcDNA3.1-COL1A1, COL1A1), and the empty pcDNA3.1 vector was applied as the negative control (oe-nc; vector). Cells were seeded (5 × 10^4^ cells/well) in 24-well plates. Transfection was performed with Lipofectamine 3000 transfection reagent (Invitrogen) according to the manufacturer’s protocol. RT–qPCR analysis was performed to assess the transfection efficiency. Forty-eight hours after transfection, cells were harvested and used in subsequent experiments.

### Adenovirus construction and infection

Both the empty adenoviral vector (Ad-sh-NC) and the Linc00511-specific small hairpin RNA (Ad-sh-linc00511) were provided by Hanbio Technology Ltd. (Shanghai, China) and transduced into A549 cells. COL1A1 overexpression lentiviral vectors (Ad-COL1A1) and its corresponding negative control vectors (Ad-NC) were provided by GenePharma (Shanghai, China). The adenovirus titer was approximately 1 × 10^10^ PFU/mL. Adenoviruses were transduced at a high multiplicity of infection (MOI; 100).

### Cell proliferation assay

A Cell Counting Kit-8 (CCK-8) assay was performed to evaluate the cell proliferation capacity. Transfected and nontransfected cells were inoculated into 96-well plates (2 × 10^3^ cells/well) and maintained in RPMI-1640 medium for 24, 48, and 72 h. Subsequently, the cells in each well were treated with 10 µL of CCK-8 reagent (Sigma–Aldrich) for 4 h. Finally, the absorbance of each well was quantified at 450 nm in a microplate reader (BioTek Instruments).

### Colony formation assay

For the colony formation assay, each group of cells was seeded into 6-well plates at a low density of 500 cells/well and cultured for 10 days [22]. Then, the cells were stained using 0.5% crystal violet solution (Sigma). After rinsing the cells to clean them, digital images were acquired under a microscope (Olympus Inc.), and visible colonies were counted.

### Apoptosis assay

The apoptosis rate was determined using an Annexin V-PE/7-AAD apoptosis detection kit (RiboBio, China). Transfected A549 and PC9 cells were inoculated into 6-well plates at a high density (2 × 10^5^ cells/well) according to the manufacturer’s instructions. After 72 h of incubation, cells were collected and stained with Annexin V PE and 7-AAD. The stained cells were examined in a FACScan flow cytometer (Agilent, China).

### Transwell assays

To assess cell invasion and migration abilities, we carried out Transwell assays. The migration assay was performed using a 24-well Transwell chamber (Cambridge, MA) according to the manufacturer’s instructions. A total of 1 × 10^5^ transfected A549 and PC9 cells were resuspended in suitable serum-free medium and then seeded separately in the upper compartments of 24-well Transwell plates. Then, 600 µL of culture medium containing 10% serum was added to the lower compartments. After 24 h of incubation, the cells on the lower surface of the membrane were fixed and stained with 1% crystal violet, and images of five random fields were then acquired under a microscope to observe and count the stained cells. For the invasion assay, 5 × 10^5^ A549 and PC-9 cells were seeded into the upper compartments containing a Matrigel (BD Biosciences)-coated membrane; the other steps were similar to those in the migration assay.

### Gene Ontology enrichment analysis

The online database TargetScan (http://www.targetscan.org/) and the Encyclopedia of RNA Interactomes (ENCORI; starBase v2.0; http://starbase.sysu.edu.cn/) were adopted to predict the binding sequences of Linc00511, miR-126-5p and miR-218-5p. Then, the overlapping miR-126-5p and miR-218-5p target genes in LUAD cells were subjected to Gene Ontology (GO) analysis, and the results were visualized with Jvenn (http://jvenn.toulouse.inra.fr/app/example.html).

### Dual luciferase reporter assay

The pmirGLO plasmid (Promega, USA) was used to construct the Linc00511 and COL1A1 wild-type luciferase reporter vectors (Linc00511-WT and COL1A1-WT) and their corresponding mutants (Linc00511-MUT and COL1A1-MUT). These luciferase reporter vectors were cotransfected with the miR-126-5p mimic, miR-218-5p mimic, or miR-NC into A549 cells. After 48 h, luciferase activity was measured with a dual luciferase reporter system (Promega, USA).

### RNA pull-down assay

MiR-126-5p, miR-218-5p, and miR-NC were biotin-labeled to generate the Bio-miR-126-5p, Bio-miR-218-5p, and Bio-NC constructs, which were transfected into cells. Forty-eight hours after transfection, cells were harvested and lysed. Cell lysates were processed with an appropriate amount of Dynabeads M-280 Streptavidin (Invitrogen, CA) for 2 h to allow streptavidin-biotin binding and were then eluted with biotin elution buffer (20 mM Tris-HCl (pH 7.4), 400 mM KCl, 0.5% NP-40, 5 mM biotin, and 80 U/mL RNase inhibitor). Finally, the biotin-coupled RNA complexes were precipitated and subjected to RT–qPCR to analyze the enrichment of the target genes.

### Western blotting

Total protein was extracted from cells with RIPA lysis buffer (Beyotime, P0013B, China) containing 50 mM Tris (pH 7.4), 150 mM NaCl, 1% Triton X-100, 1% sodium deoxycholate, 0.1% SDS, and various inhibitors (sodium orthovanadate, sodium fluoride, EDTA, leupeptin, etc.), and then quantified with a BCA Protein Assay Kit (Thermo Scientific, USA). A 30 µg denatured protein sample was separated by 10–12% SDS–PAGE and was then transferred to PVDF membranes (Millipore). After blocking in 5% nonfat milk for 2 h at room temperature, the membranes were incubated with primary antibodies specific for COL1A1 (1:1000, Abcam, UK), PI3K (1:500, Abclonal Technology, USA), p-PI3K (1:500, Abclonal Technology, USA), AKT (1:500, Abclonal Technology, USA), and p-AKT (1:500, Abclonal Technology, USA) overnight at 4 °C. Thereafter, incubation with the secondary antibody was conducted for an additional 1 h at room temperature. Finally, an ECL reagent was employed for protein visualization (Millipore, USA). GAPDH was employed as the internal control.

### RT–qPCR

We utilized TRIzol reagent (Gibco-BRL, USA) to selectively extract total RNA from tissues or cells, and first-strand cDNA was then synthesized using a PrimeScript™ RT Master Mix Kit (TaKaRa, Shiga, Japan). RT–qPCR was conducted with a commercial SYBR Premix Dimer Eraser Kit (Takara, China), and the target mRNA and miRNA levels were measured in an ABI 7500 PCR instrument (Applied Biosystems) according to the manufacturer’s recommendations. U6 (for miR-126-5p and miR-218-5p) and GAPDH (for Linc00511 and COL1A1) were used as the internal controls. The fold changes in the expression of the target genes were measured by the 2^−∆∆Ct^ method. The sequences of the primers used in this study are shown in Table 1.

**Table 1:** Sequences for real-time quantitative polymerase chain reaction

Gene	Sequence	
	Forward	Reverse
Linc00511	5′-CTAACAAGAGGGTAAGTGTCAG-3′	5′-AAGTCGACAACCCCATCGTTAC-3′
miR-126-5p	5′-GGTATAATCCGCCGCTTAGCTGCC-3′	5′-GTGCAGGGTTGCAAGGT-3′
miR-218-5p	5′-AACACGAACTAGATTGGTACA-3′	5′-AGTCTCAGGGTCCGAGGTATTC-3′
COL1A1	5′-GAGGGCCAAGACGAAGACATC-3′	5′-CAGATCACGTCATCGCACAAC-3′
GAPDH	5′-AATGGATTTGGACGCATTGGT-3′	5′-TTTGCACTGGTACGTGTGTTGAT-3′
U6	5′-CTCGCTTCGGCAGCACA-3′	5′-AACGCTTCACGAATTTGCGT-3′

### Tumor xenograft models

All animal studies were performed strictly according to a protocol approved by the Ethics Committee of Shengjing Hospital of China Medical University. BALB/c nude mice (male, 4 to 8 weeks old, weighing 18–20 g) were obtained from Shanghai SLAC Laboratory Animal Co., Ltd. (Shanghai, China). For each group (six male mice), A549 cells transfected with Ad-sh-NC, Ad-NC, Ad-sh-linc00511 or Ad-COL1A1 were resuspended in PBS and then inoculated subcutaneously into BALB/c mice at a viable cell density of 1 × 10^7^ cells/mL. Mice were randomly divided into three groups: the Ad-sh-NC group, Ad-sh-linc00511 + Ad-NC group and Ad-sh-linc00511 + Ad-CLO1A1 group. One week after injection, tumor size and volume were monitored once every four days by measuring the tumors in two dimensions—width (W) and length (L)—using an electronic caliper. Tumor volumes were estimated with the following formula: V = (W^2^ × L)/2. All mice were euthanized on Day 27 with a CO_2_ overdose, and the xenograft tumors were excised.

### Statistical analysis

Study measurements are expressed as the means ± SDs, and all assays were performed in triplicate. Statistical differences between two or among multiple groups were determined using Student’s *t* test or one-way ANOVA, respectively. Statistical analyses of gene or protein expression data were carried out via SPSS 21.0 statistical software (SPSS Inc., Chicago, IL), and graphs were generated in Prism software (GraphPad, Version 8.1.1, CA, USA). A *P* value of less than 0.05 was considered the criterion for statistical significance.

## Results

### The Linc00511 level is increased in lung adenocarcinoma tissues and cells

**Fig. 1:**
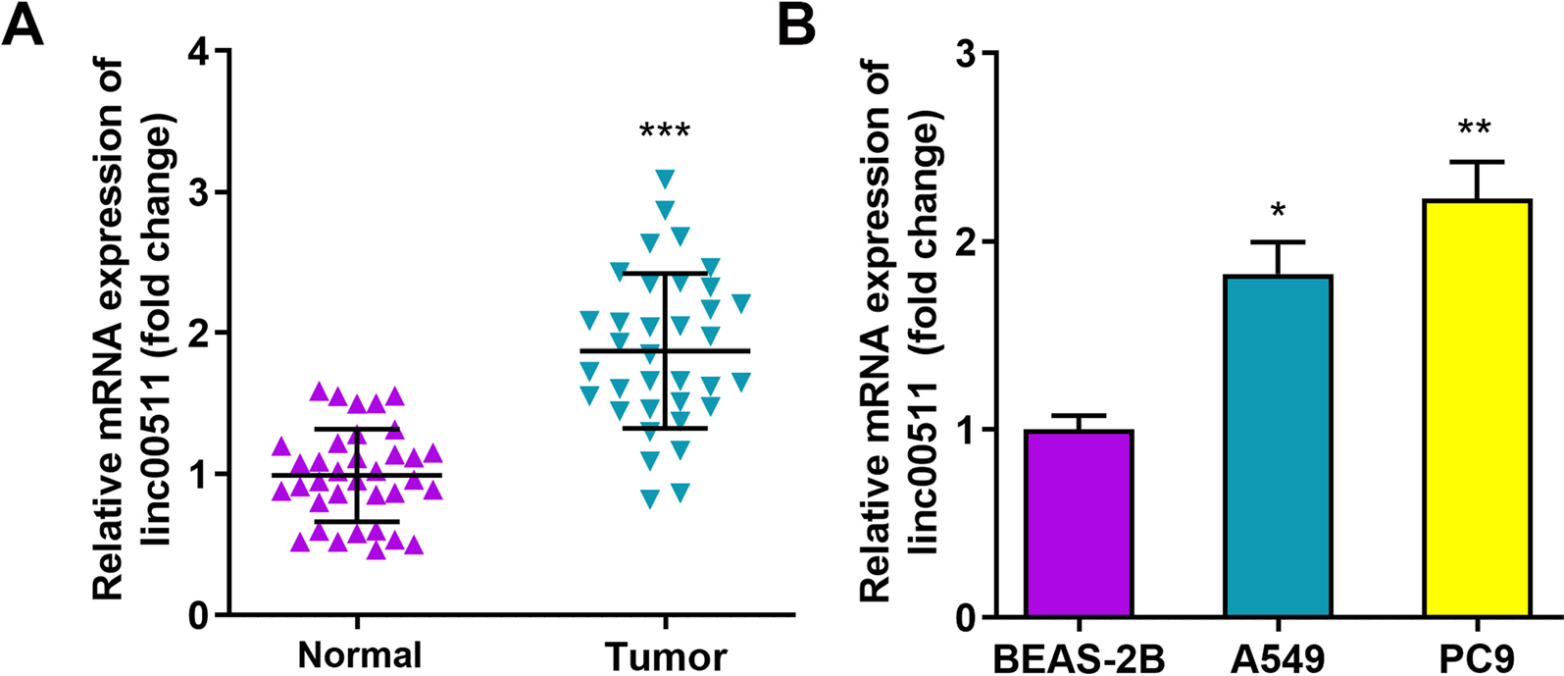
Linc00511 is upregulated in lung adenocarcinoma tissues and cells. **A** The relative expression of Linc00511 in lung adenocarcinoma tissues and adjacent normal tissues (n = 35) was measured by RT–qPCR. **B** RT–qPCR was used to measure the relative Linc00511 levels in BEAS-2B, A549 and PC9 cells. *P < 0.05, **P < 0.01, ***P < 0.001 versus normal or BEAS-2B cells

To verify whether Linc00511 is associated with LUAD, we first detected abnormal Linc00511 expression in 35 paired LUAD tissues and matched nontumor surgical lung tissues by RT–qPCR. The data revealed that Linc00511 had high expression in LUAD tissues compared with the paired normal tissues (Fig. 1A). In addition, the relative Linc00511 expression level was significantly increased in A549 and PC9 cells compared with BEAS-2B bronchial epithelial cells (Fig. 1B). These results suggest that the differential expression of Linc00511 might be closely associated with LUAD progression and tumorigenesis.

### Silencing Linc00511 suppresses cell proliferation, migration and invasion and promotes apoptosis

**Fig. 2:**
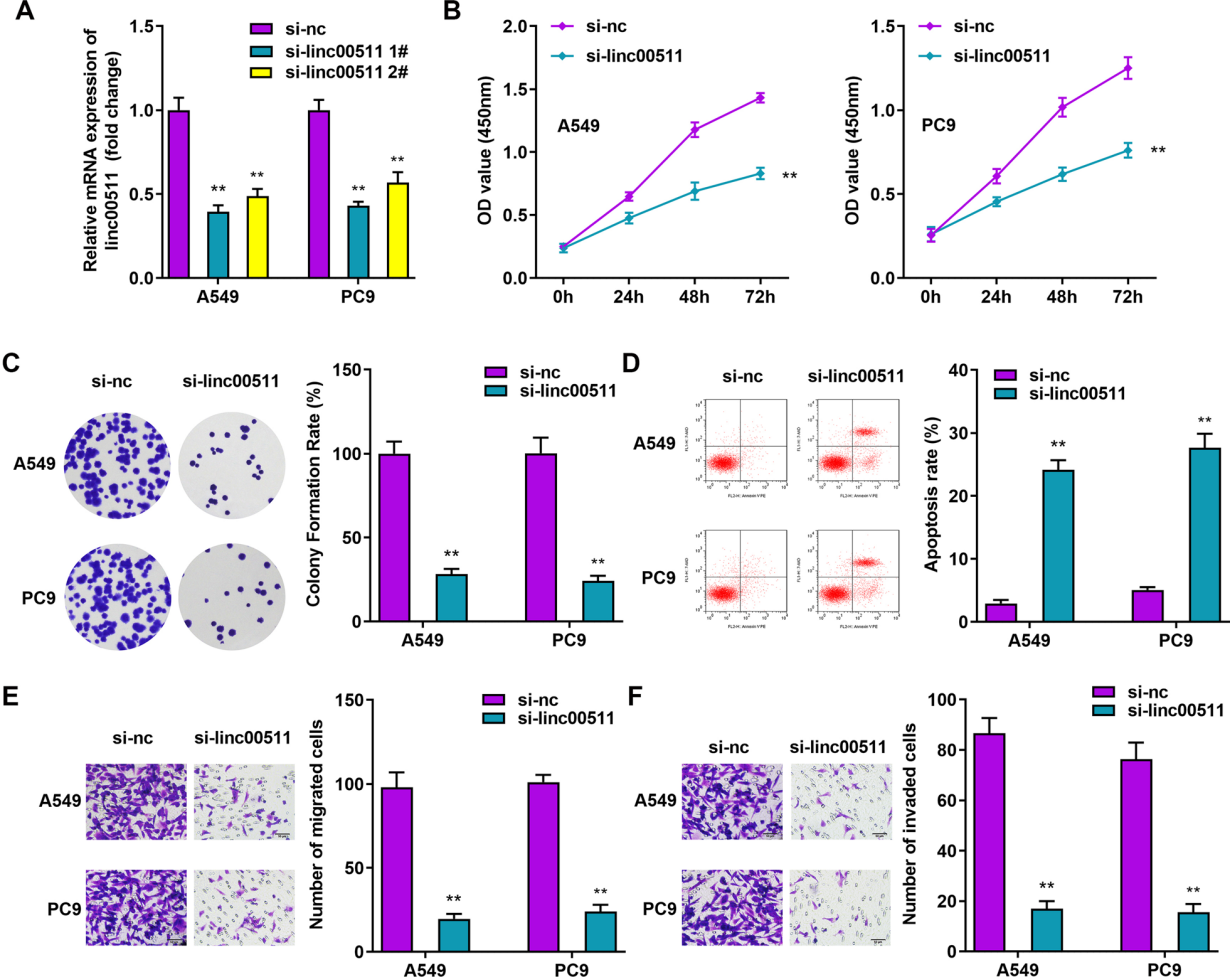
Linc00511 downregulation inhibits LUAD cell proliferation, migration, and invasion and promotes LUAD cell apoptosis. **A** The Linc00511 knockdown efficiency in A549 and PC9 cells was determined by RT–qPCR. The proliferation and viability of A549 and PC9 cells after silencing Linc00511 was evaluated by CCK-8 (**B**) and colony formation assays (**C**). **D** The impact of Linc00511 silencing on A549 and PC9 cell apoptosis was assessed by flow cytometric analysis. Transwell assays were used to verify the changes in the migratory (**E**) and invasive (**F**) abilities of A549 and PC9 cells after Linc00511 knockdown. *P < 0.05, **P < 0.01, ***P < 0.001 versus si-nc, Scale bars, 50 μm

There are numerous previous reports in the literature demonstrating that Linc00511 could regulate tumor cell proliferation, apoptosis, migration, and invasion [23, 24]. To determine whether Linc00511 plays a positive role in the pathogenesis of LUAD, we transfected A549 and PC9 cells with si-linc00511 to specifically silence Linc00511 expression. Here, we designed loss-of-function assays to evaluate the effects of Linc00511 on the biological behaviors of tumor cells. The RT–qPCR results showed that both si-linc00511 #1 and si-linc00511 #2 exhibited good knockdown efficiency, suggesting that the cells were efficiently transfected (Fig. 2A). However, si-linc00511 #1 significantly downregulated the relative Linc00511 expression level by as much as 64%. Therefore, we selected si-linc00511 #1 for the next experiments. The colony formation and CCK-8 assays showed that downregulation of Linc00511 significantly decreased the cell proliferation and colony formation abilities of both A549 and PC9 cells relative to those in the blank control groups (Fig. 2B, C). Flow cytometric analysis showed that Linc00511 knockdown markedly elevated apoptosis in PC9 and A549 cells, which confirmed the antitumor roles of si-linc00511 (Fig. 2D). However, evident decreases in the migration and invasion abilities of A549 and PC9 cells transfected with si-linc00511 were observed compared with those of control cells (Fig. 2E, F). These results suggest that upregulation of Linc00511 enhances the malignancy of LUAD, indicating that Linc00511 plays a vital role in the development of LUAD as a pro-oncogenic factor.

### Overexpression of Linc00511 contributes to maintaining malignant biological behaviors of LUAD cells

**Fig. 3:**
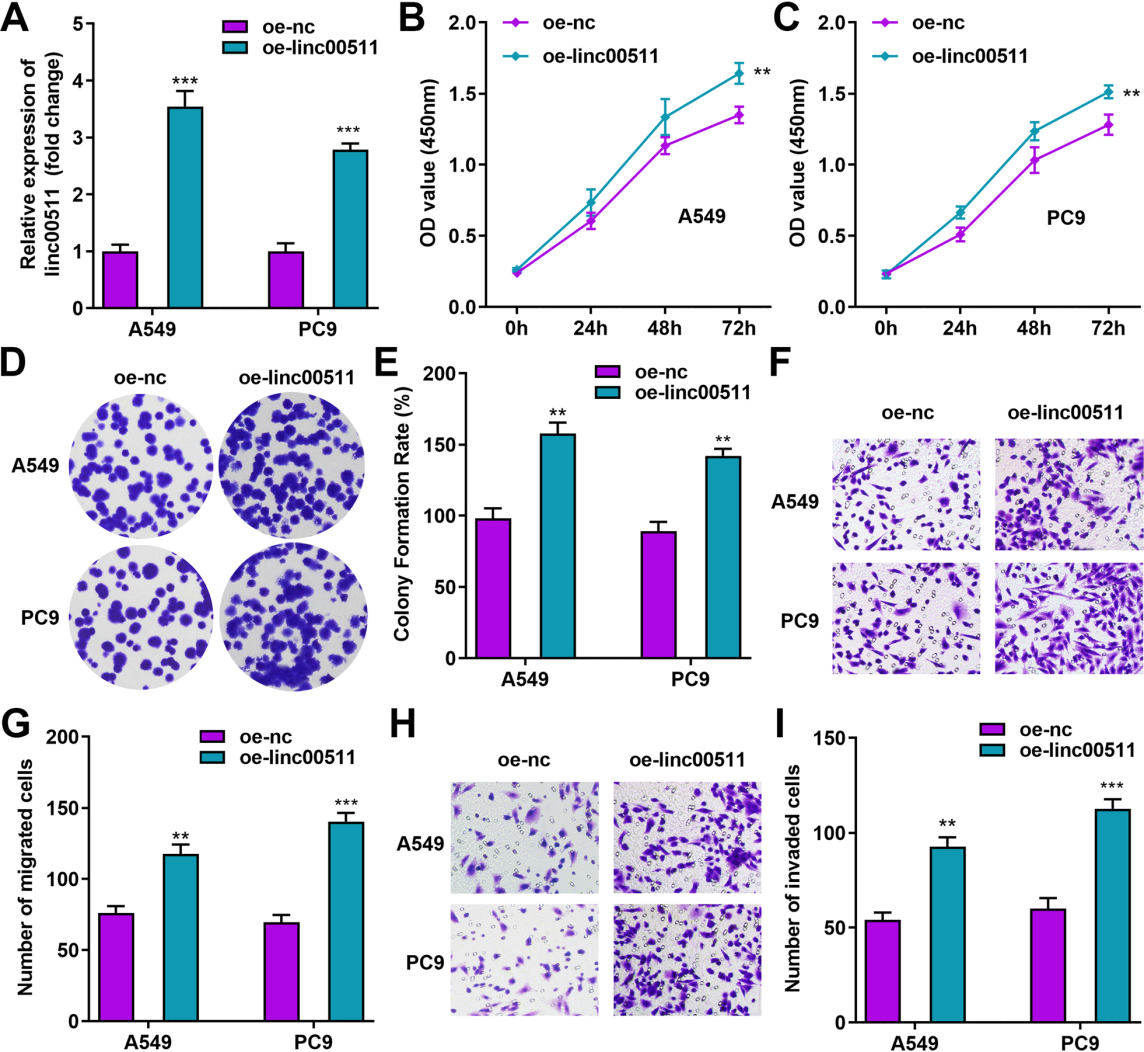
Linc00511 overexpression enhances the proliferation, migration and invasion of LUAD cells. **A** The relative expression of Linc00511 in A549 and PC9 cells transfected with oe-nc and oe-lin00511 was determined by RT–qPCR. **B–E** The proliferation ability of A549 and PC9 cells after overexpression of Linc00511 was examined by a CCK-8 assay (**B**, **C**) and a colony formation assay (**D**, **E**). **F**–**I** Transwell assays were performed to determine the changes in the migratory (**F**, **G**) and invasive (**H**, **I**) abilities of A549 and PC9 cells after Linc00511 overexpression. **P < 0.01, ***P < 0.001 versus oe-nc

After determining the effects of Linc00511 silencing in LUAD cells, we further generated LUAD cells with overexpression of Linc00511 by introducing pcDNA3.1-Linc00511 (labeled oe-linc00511; with empty vector (labeled oe-nc) as the control) into A549 and PC9 cells. As shown in Fig. 3A, transfection of the Linc00511 overexpression vector effectively increased the relative level of Linc00511 in both LUAD cell lines. Furthermore, the CCK-8 assay showed that Linc00511 overexpression substantially enhanced the proliferation and viability of A549 and PC9 cells (Fig. 3B, C). Similarly, for both A549 and PC9 cells, the colony formation rate was higher in the oe-linc00511 group than in the oe-nc group (Fig. 3D, E). Based on Transwell assays, we also observed that Linc00511 overexpression enhanced the invasive and migratory abilities of A549 and PC9 cells, as evidenced by the increased number of migrating and invading cells of both cell lines (Fig. 3F–I). The above results imply that overexpression of Linc00511 could enable invasive LUAD cells to acquire enhanced proliferative and metastatic abilities.

### MiR-126-5p and miR-218-5p act as targets of Linc00511

**Fig. 4:**
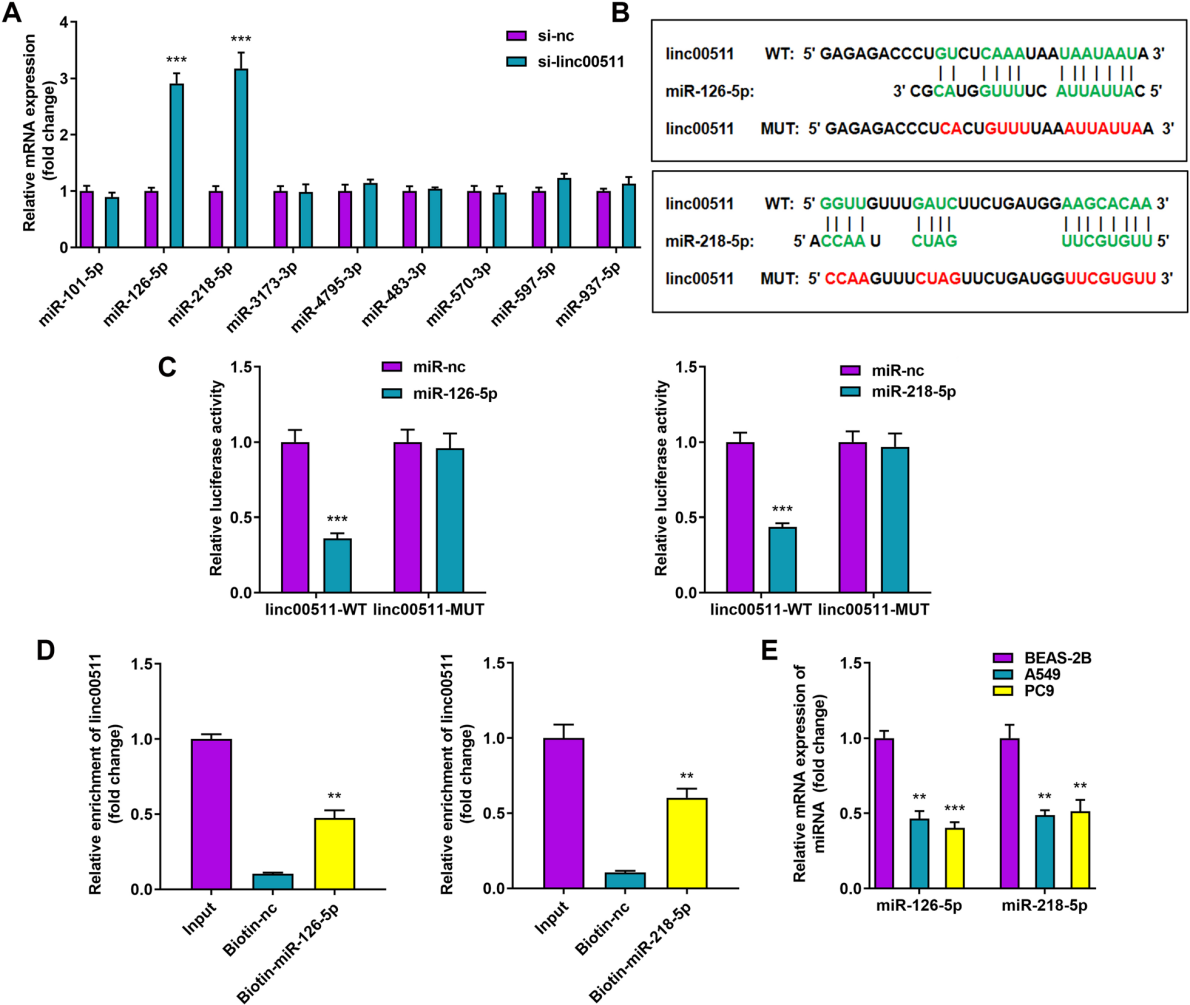
Linc00511 interacts with miR-126-5p and miR-218-5p. **A** After Linc00511 knockdown, the levels of nine differentially expressed miRNAs in lung adenocarcinoma were determined by RT–qPCR. **B** The potential targeting sequences of miR-126-5p and miR-128-5p in Linc00511 were predicted, and a schematic of the wild-type and mutant Linc00511 constructs is shown. **C** Dual luciferase reporter vectors were used to test whether Linc00511 directly binds to miR-126-5p and miR-218-5p. Luciferase activity was measured in A549 cells cotransfected with Linc00511-WT or Linc00511-MUT and the miR-126-5p mimic or miR-218-5p mimic. **D** An RNA pulldown assay revealed the interactions of Linc00511 with miR-126-5p and miR-218-5p. **E** Measurement of miR-126-5p and miR-218-5p mRNA levels in BEAS-2B, A549, and PC9 cells. **P < 0.01, ***P < 0.001 versus the si-nc, miR-NC, BEAS-2B, or Biotin-nc group

Linc00511 could perform its function by sponging related miRNAs, thereby influencing the degradation or translation of their target genes. To further discover the mechanism of Linc00511 in LUAD, we first identified nine candidate miRNAs stably expressed in LUAD (miR-101-5p, miR-126-5p, miR-218-5p, miR-3173-3p, miR-4795-3p, miR-483-3p, miR-570-3p, miR-597-5p and miR-937-5p) through a systematic literature screen and bioinformatics tools. After knockdown of Linc00511, the levels of these nine candidate miRNAs were verified by RT–qPCR to identify the differentially expressed miRNAs. Further results showed that two miRNAs (miR-126-5p and miR-218-5p) were significantly differentially expressed after Linc00511 was silenced (Fig. 4A). It was preliminarily hypothesized that Linc00511 has the potential to regulate miR-126-5p and miR-218-5p. We performed bioinformatic analysis of Linc00511 with the TargetScan database and found that miR-126-5p and miR-218-5p have several potential binding sites for Linc00511 (Fig. 4B). To confirm this prediction, we performed a dual luciferase reporter assay in A549 cells. As shown in Fig. 3C, miR-126-5p and miR-218-5p decreased the luciferase activity of Linc00511-WT but had no influence on the luciferase activity of Linc00511-MUT, implying that Linc00511 can bind directly to miR-126-5p and miR-218-5p (Fig. 4C). Likewise, the RNA pulldown assay revealed that Linc00511 was enriched in the Biotin-miR-126-5p-WT and Biotin-miR-218-5p-WT samples but not in the Biotin-miR-126-5p-MUT, Biotin-miR-218-5p-MUT, and Biotin-nc samples (Fig. 4D). To verify whether miR-126-5p or miR-218-5p is associated with LUAD progression, we examined the miR-126-5p and miR-218-5p expression levels in LUAD cells. The RT–qPCR data also showed that the relative levels of miR-126-5p and miR-218-5p were markedly lower in A549 and PC-9 cells than in normal cells (Fig. 4E) and that the trends in their expression were opposite that of Linc00511 in LUAD. Collectively, these data suggested that Linc00511 acts as a molecular sponge for miR-126-5p and miR-218-5p in LUAD to inhibit their function.

### Linc00511 upregulates COL1A1 expression through competitive binding to miR-126-5p or miR-218-5p

**Fig. 5:**
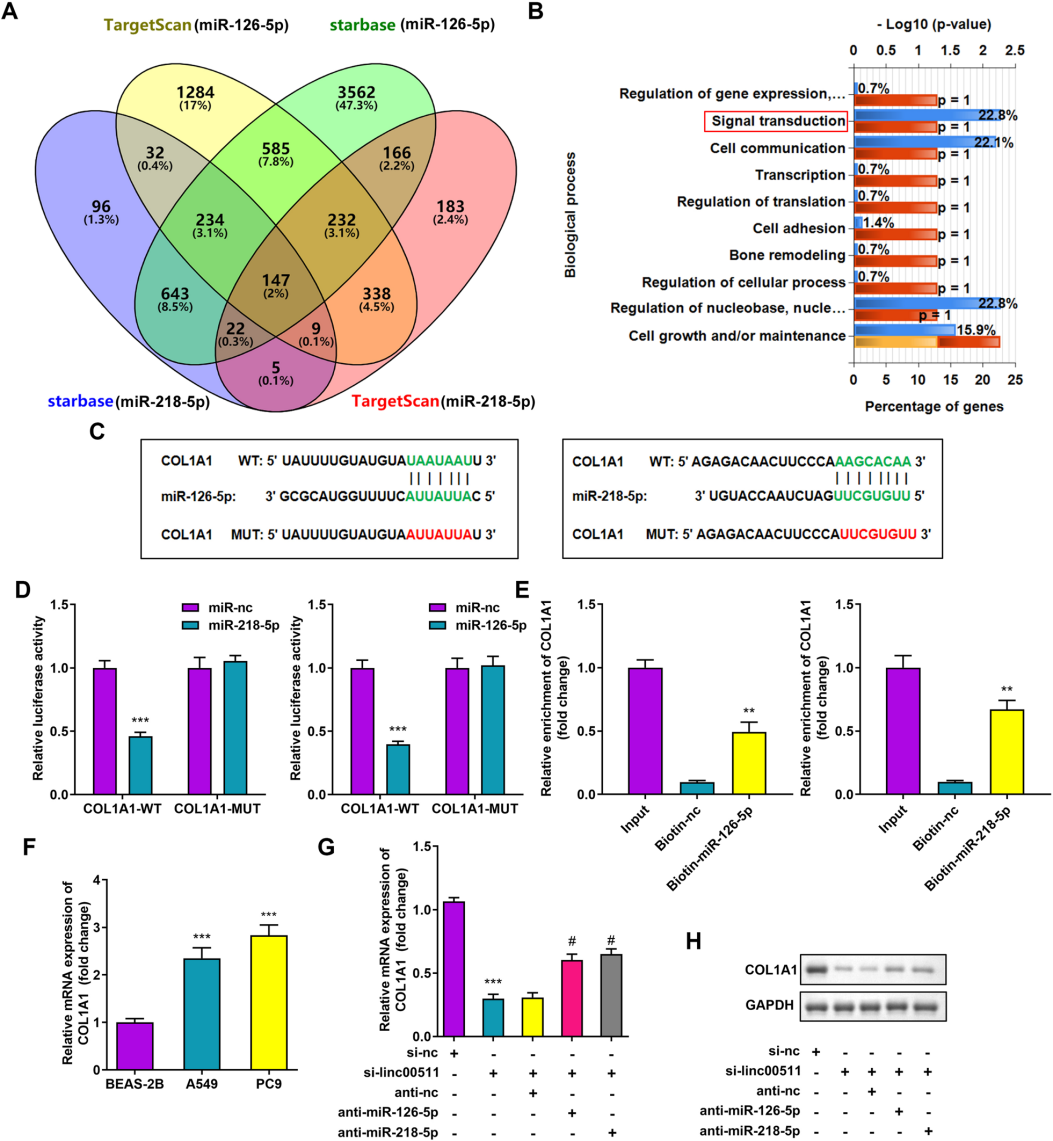
Linc00511 regulates COL1A1 expression by targeting miR-126-5p or miR-218-5p. **A** The Venn diagram shows the overlapping genes coregulated by miR-126-5p and miR-218-5p that were predicted by the TargetScan and starBase databases. **B** Gene ontology (GO) enrichment analysis of miR-126-5p and miR-218-5p target genes. **C** Prediction of miR-126-5p and miR-218-5p binding sites in the COL1A1 mRNA 3’-UTR. **D** Dual luciferase reporter vectors were used to verify the interactions of miR-126-5p and miR-218-5p with COL1A1. **E** An RNA pulldown assay confirmed the relationships between COL1A1 and both miR-126-5p and miR-218-5p. **F** The relative COL1A1 expression levels were examined in BEAS-2B, A549 and PC9 cells by RT–qPCR. **G** COL1A1 mRNA and **H** protein expression in A549 cells transfected with si-linc00511, anti-nc + si-linc00511, anti-miR-218-5p + si-linc00511, or anti-miR-126-5p + si-linc00511. **P < 0.01, ***P < 0.001 versus the miR-NC, BEAS-2B or Biotin-nc group; ^#^*P* < 0.05 versus the si-linc00511 + anti-nc group

To further analyze the regulatory network of Linc00511, the downstream targets of miR-126-5p and miR-218-5p were predicted using the TargetScan web server and starBase v2.0. The intersection of the mRNAs predicted by both databases was determined, and Venn diagrams of those mRNAs were generated (Fig. 5A). Functional enrichment analysis of the overlapping mRNAs showed that these mRNAs mediated many biological processes, mainly signal transduction, intercellular communication, nucleobase regulation, and cell growth or maintenance (Fig. 5B). Bioinformatic analysis showed that the COL1A1 3’-UTR contains binding sites for miR-126-5p and miR-218-5p (Fig. 5C), consistent with previous studies [25]. To verify the endogenous mutual interactions between COL1A1 and both miR-126-5p and miR-218-5p, we constructed COL1A1 3’-UTR WT and COL1A1 3’-UTR MUT plasmids. The dual luciferase reporter assay results showed that the luciferase activity of COL1A1 3’-UTR WT was significantly reduced after cotransfection with the miR-126-5p mimic or miR-218-5p mimic (*P* < 0.001), whereas COL1A1 3’-UTR MUT was not affected (Fig. 5D). Similarly, the RNA pulldown assay confirmed the binding relationships between COL1A1 and both miR-126-5p and miR-218-5p (Fig. 5E). In addition, we verified the upregulation of COL1A1 expression in LUAD cell lines, with PC9 cells exhibiting the highest COL1A1 expression (Fig. 5F). To further explore the existence of specific crosstalk among Linc00511, COL1A1 and miR-126-5p or miR-218-5p, we transfected si-nc, si-linc00511, anti-nc + si-linc00511, anti-miR-218-5p + si-linc00511 or anti-miR-126-5p + si-linc00511 into A549 cells. The RT–qPCR and western blot results showed that knockdown of Linc00511 significantly suppressed the relative COL1A1 mRNA and protein expression but miR-126-5p or miR-218-5p downregulation rescued the Linc00511 silencing-mediated reductions in COL1A1 mRNA and protein expression (Fig. 5G, H). These results suggested that COL1A1 is a target of miR-126-5p and miR-218-5p and that Linc00511 can regulate COL1A1 expression in LUAD via miR-126-5p or miR-218-5p.

### COL1A1 overexpression reverses the effect of Linc00511 silencing on LUAD progression

**Fig. 6:**
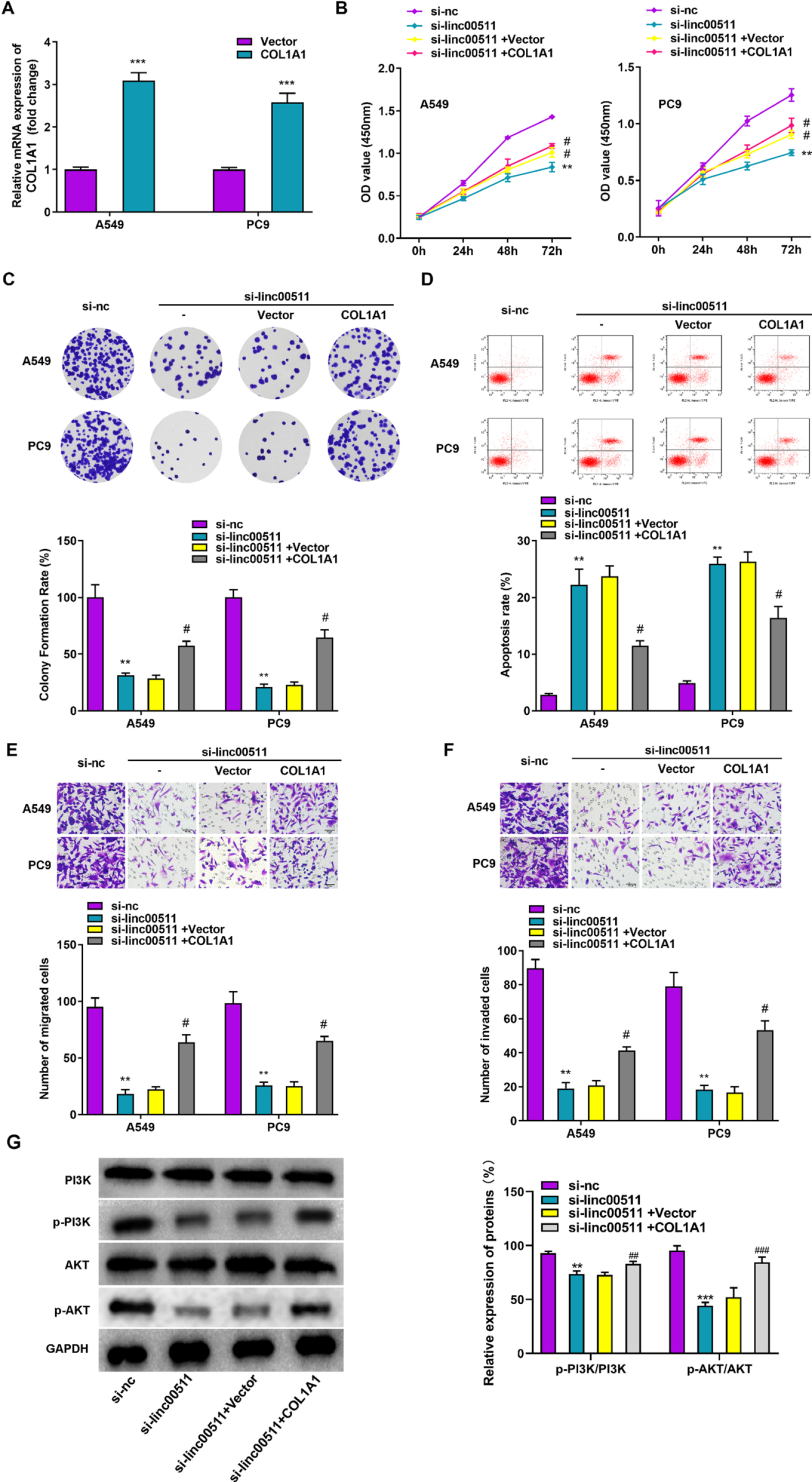
Linc00511 promotes the proliferation, migration and invasion of lung adenocarcinoma cells by regulating COL1A1 expression. **A** Measurement of the COL1A1 overexpression efficiency by RT–qPCR in A549 and PC9 cells. **B**–**F** Cell proliferation (**B**), colony formation (**C**), apoptosis (**D**), migration (**E**) and invasion (**F**) were evaluated in A549 and PC9 cells transfected with si-nc, si-Linc00511, si-Linc00511 + vector or si-Linc00511 + COL1A1. **G** The PI3K, phosphorylated PI3K (p-PI3K), AKT, and phosphorylated AKT (p-AKT) levels were determined by western blot analysis in LUAD cells with Linc00511 silencing or forced COL1A1 expression. * *P* < 0.05, ** *P* < 0.01, ***P < 0.001 versus the si-nc group; ^#^ *P* < 0.05, ^##^ *P* < 0.01, ^###^*P* < 0.001 versus the si-linc00511 + vector group

To confirm whether COL1A1 can interact with Linc00511 to participate in LUAD progression, the COL1A1 overexpression vector (COL1A1) or empty vector was transfected into A549 and PC9 cells. The RT–qPCR results showed that overexpressing COL1A1 increased the relative COL1A1 mRNA expression levels by more than 2.5-fold and that the transfection efficiency was higher in A549 cells (Fig. 6A). We next performed rescue experiments in which A549 and PC9 cells were transfected with si-nc, si-linc00511, si-linc00511 + vector, or si-linc00511 + COL1A1. CCK-8 and colony formation assays showed that compared to that of cells in the si-nc group, the proliferation capacity of cells in the si-linc00511 and si-linc00511 + vector groups was significantly reduced, while this effect was reversed by upregulation of COL1A1 (Fig. 6B, C). In addition, the promoting effects of Linc00511 knockdown on apoptosis, migration, and invasion were reversed by COL1A1 overexpression (Fig. 6D–F). Several studies have indicated that the PI3K/AKT pathway is aberrantly activated in lung cancer and controls core cellular functions [26]. Both lncRNAs and COL1A1 are associated with the regulation of miRNA expression or PI3K/AKT signaling pathway activity in cancer [27]. The PI3K and AKT phosphorylation levels were reduced in the si-linc00511 group compared to the si-nc group, while COL1A1 overexpression partially rescued the reductions in the p-PI3K and p-AKT protein levels caused by Linc00511 depletion (Fig. 6G). Our results suggested that Linc00511 silencing may retard LUAD progression by regulating COL1A1 and that Linc00511 regulation involves activation of the PI3K/AKT signaling pathway in LUAD cells.

### Linc00511 promotes tumor growth in vivo

**Fig. 7:**
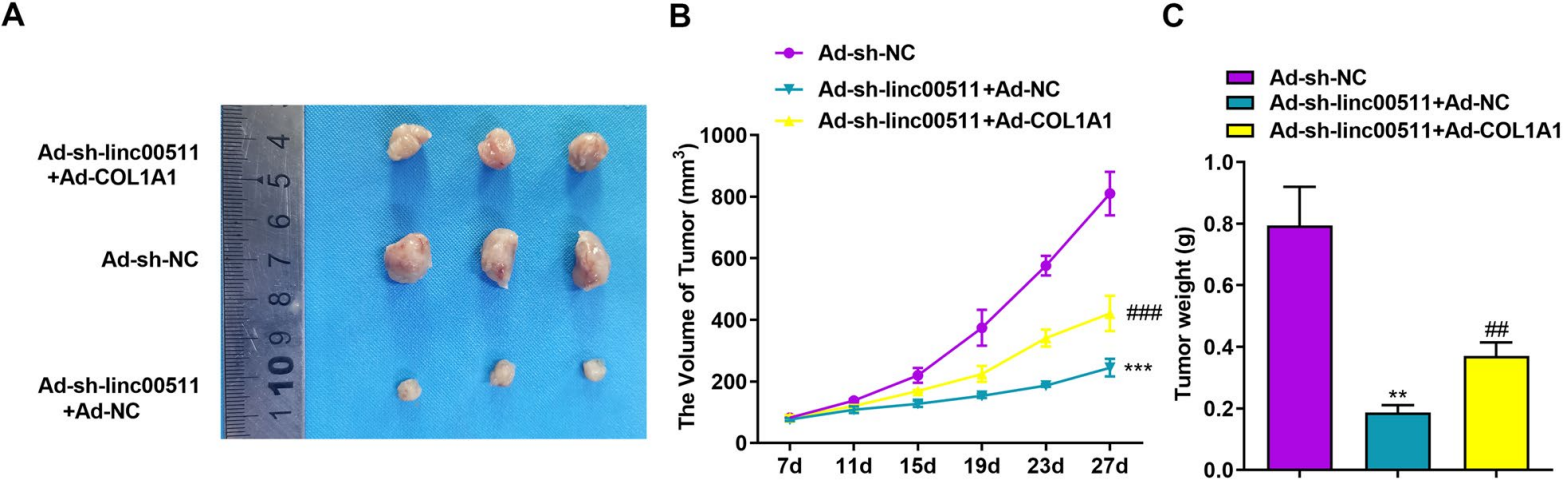
Knockdown of Linc00511 expression inhibits tumor growth ***in vivo***. **A** Representative images of tumors in mice injected with Ad-sh-NC/A549, Ad-sh-linc00511 + Ad-NC/A549 and Ad-sh-linc00511 + Ad-COL1A1/A549. **B** Tumor growth curves of mice after injection of Ad-sh-NC/A549, Ad-sh-linc00511 + Ad-NC/A549 and Ad-sh-linc00511 + Ad-COL1A1/A549. **C** Tumor weights were measured at the experimental endpoint. ^##^*P* < 0.01, ^###^*P* < 0.001 versus the Ad-sh-linc00511 + Ad-NC group; **P < 0.01, ***P < 0.001 versus the Ad-sh-NC group

To further elucidate how Linc00511 affects tumor growth in vivo, we subcutaneously injected A549 cells stably transfected with Ad-sh-NC, Ad-sh-linc00511 + Ad-NC or Ad-sh-linc00511 + Ad-COL1A1 into the right flank of nude mice to establish a xenograft model. After injection, the tumor volume and size were measured once every four days. After 27 days, the tumor volume and weight in the Ad-sh-linc00511 + Ad-NC group were decreased compared with those in the Ad-sh-NC group due to downregulation of Linc00511 (Fig. 7A–C). However, as the data showed for the Ad-sh-linc00511 + Ad-COL1A1 group, COL1A1 overexpression partially reversed this reduction. The above experimental results indicated that silencing Linc00511 expression could inhibit malignant progression in nude mice and further verified the value of the Linc00511/COL1A1 axis in vivo.

## Discussion

Asymptomatic growth and long-distance migration are important obstacles to improving the poor prognosis of LUAD; therefore, there is a crucial need to explore the potential regulatory mechanisms and to find new molecular therapeutic targets for LUAD. Numerous reports have shown that abnormally expressed lncRNAs are involved in several cancer-related processes, such as cell proliferation, apoptosis, metastasis, and metabolism. In addition, certain lncRNAs have been identified as specific to developmental stages, disease types, and tissues. Competing endogenous RNAs (ceRNAs) competitively bind to the same miRNA, reversing the inhibitory effect of the miRNA on its target mRNA; this phenomenon is called the ceRNA mechanism. Correspondingly, the discovery of ceRNAs has enhanced our knowledge of the molecular mechanisms underlying cancer development and progression. LncRNAs acting as ceRNAs have structures similar to those of mRNAs and act as target genes for miRNAs to regulate the expression of protein-coding genes [28]. Current studies indicate that lncRNAs can be involved in LUAD development and progression through ceRNA regulatory mechanisms. For example, LncRNA-XIST can promote CBLL1 expression by binding to miR-212-3p, which in turn promotes NSCLC cell proliferation and invasion [29]. Therefore, further study of the links between tumorigenesis and lncRNAs will contribute to a better understanding of cancers.

Linc00511, a newly discovered lncRNA, is involved in the development of numerous cancers through the ceRNA mechanism and is associated with poor prognosis in cancers. In gastric cancer, Linc00511 promotes progression by sponging miR-124-3p and thus targeting PDK4 [30]. Linc00511 can suppress miR-185-3p expression and target E2F1 protein expression, which in turn promotes osteosarcoma cell proliferation and migration [11]. Similar results were observed in cervical cancer, in which Linc00511 could act as a ceRNA to regulate the miR-324-5p/DRAM1 axis, which had positive associations with tumor malignancy and poor prognosis [31]. These results provide direct evidence for the potential oncogenic role of Linc00511 in tumors. Previously, it was reported that Linc00511 was highly expressed in LUAD tissues and cells and was positively correlated with poor patient prognosis. Furthermore, Linc00511 was reported to act as a ceRNA of miR-625-5p to increase PKM2 expression and promote LUAD cell proliferation, migration, and invasion [13]. We verified that Linc00511 expression is upregulated in LUAD. We also found that knockdown of Linc00511 inhibited LUAD cell proliferation, migration, and invasion in vitro. In fact, gain-of-function studies revealed that increased Linc00511 expression enhanced the malignant phenotype of LUAD cells.

Determining the molecular mechanism of Linc00511 in LUAD will facilitate the exploration of effective therapeutic strategies. Numerous studies on lncRNAs have proven that lncRNAs can simultaneously interact with multiple miRNAs. With the help of the TargetScan database, we found that miR-126-5p and miR-218-5p have binding sites for Linc00511. Previous reports have suggested that miR-126-5p is expressed at low levels in lung adenocarcinoma tissues and significantly correlates with patient prognosis. miR-218-5p was found to be significantly downregulated in numerous cancers, including lung, liver, gastric, and bladder cancers, thus acting as a tumor suppressor miRNA [32–35]. These studies strongly suggest that miR-126-5p and miR-218-5p may play a key regulatory role in LUAD development. Here, our findings showed that miR-126-5p and miR-218-5p were downregulated in LUAD tissues and cell lines. After knockdown of Linc00511, the expression of miR-126-5p and miR-218-5p was upregulated. The presence of miR-126-5p or miR-218-5p inhibitors reversed the effects of si-linc00511. Our study clearly demonstrated the inhibitory effect of Linc00511 on both miR-126-5p and miR-218-5p. In brief, Linc00511 participates in crosstalk with both miRNAs.

MiRNAs can reduce gene expression by base-pairing with the 5’-UTR or 3ʹ-UTR of their target mRNAs. By further exploring the downstream targets of Linc00511/miR-126-5p or miR-218-5p, we found using TargetScan and the starBase database that COL1A1 directly targets miR-126-5p and miR-218-5p, consistent with earlier studies [25]. Preclinical studies have shown that COL1A1 may be a biomarker for poor prognosis in gastric cancer [36]. In addition, COL1A1 may be a reliable therapeutic target for hepatocellular lesions and metastases [37]. COL1A1 has also been reported to be associated with hypoxia in NSCLC [38]. Our data suggested that overexpression of COL1A1 reversed the inhibitory effect of Linc00511 knockdown on LUAD cell migration and invasion in vitro. Therefore, we found that Linc00511 can coregulate COL1A1-mediated proliferation and metastasis in cooperation with one or more miRNAs. In fact, additional evidence for this hypothesis was found in the work of Zhao et al. [39], where COL1A1 expression was correlated with immune infiltration levels in LUAD. At the same time, COL1A1 can be regulated by the inflammatory response, and its interaction with CD276 can be used to reveal the correlation between COL1A1 expression and the potential mechanism of poor prognosis of LUAD. However, this mechanism needs further study. Another study on COL1A1 showed that regulation of the PI3K-AKT signaling pathway in cancer was associated with higher COL1A1 expression levels [27]. AKT is activated via PI3K phosphorylation and is subsequently involved in a variety of cellular processes, including cell proliferation, inflammation, cell survival and glucose metabolism [40]. Inhibition of the PIK3/AKT pathway is accompanied by increased apoptosis and decreased invasiveness of cancer cells. The role of Linc00511 as an oncogene driving PI3K/AKT signaling was demonstrated in the work of Wang et al. [41] In our study, we confirmed that the PIK3/AKT pathway is activated in LUAD, since the p-PI3K and p-AKT levels were increased by Linc00511. Mechanistically, upregulation of COL1A1 by Linc00511 led to enhanced PIK3/AKT pathway activity. From another important aspect, this finding revealed the reason that Linc00511 induces malignant cell progression in LUAD. Finally, we validated the effect of Linc00511 on lung adenocarcinoma development in vivo in an animal model. The results verified that knockdown of Linc00511 inhibited tumor growth and metastasis in nude mice, while COL1A1 overexpression partially counteracted the tumor suppression mediated by Linc00511 silencing. These encouraging results confirmed the specific mechanism by which Linc00511 acts as a ceRNA in LUAD (Additional file 1).

## Conclusions

In conclusion, this study demonstrated the potential mechanism of overexpressed Linc00511 as an oncogene in lung adenocarcinoma via construction of a lncRNA–miRNA–mRNA network. Our study identified a high Linc00511 level in LUAD and found that it was correlated with the malignant phenotype of LUAD cells. Second, bioinformatic analysis confirmed the targeted binding interaction of Linc00511 with miR-126-5p and miR-218-5p. Functionally, inhibition of miR-126-5p and miR-218-5p counteracted the effect of si-linc00511. Subsequently, we selected COL1A1 as a common target of miR-126-5p and miR-218-5p and validated the effectiveness of the Linc00511/miR-126-5p/miR-218-5p/COL1A1 axis in LUAD progression by rescue experiments. The increased aggressiveness of LUAD was also partly attributed to activation of the PIK3/AKT pathway via the Linc00511/COL1A1 axis. These results demonstrate the specific mechanism by which Linc00511 functions as a ceRNA sponge of miR-126-5p/miR-218-5p to promote COL1A1-mediated LUAD proliferation and metastasis and provide a new direction for molecular therapeutic strategies for LUAD.

## Supplementary Information

**Additional file 1.** Original results of western blot assays in the laboratory.

## Data Availability

The data that support the findings of this study are available from the corresponding author upon reasonable request.

## Abbreviations

LUAD: Lung adenocarcinoma
lncRNAs: Long noncoding RNAs
RT–qPCR: Real-time quantitative polymerase chain reaction
NSCLC: Non-small-cell lung cancer
ceRNA: Competing endogenous RNA
miRNAs: MicroRNAs
COL1A1: Collagen type I alpha 1 chain
PI3K/AKT: Phosphatidylinositol 3-kinase/AKT
si-nc: siRNA control
CCK-8: Cell Counting Kit-8
GO: Gene Ontology

## References

[1] Pan J, Fang S, Tian H, Zhou C, Zhao X, Tian H (2020). lncRNA JPX/miR-33a-5p/Twist1 axis regulates tumorigenesis and metastasis of lung cancer by activating Wnt/β-catenin signaling. Mol Cancer.

[2] Barta JA, Powell CA, Wisnivesky JP (2019). Global epidemiology of lung cancer. Ann Glob Health.

[3] Ma D, Liu H, Qin Y, Li D, Cui Y, Li L (2020). Circ_0007142/miR-186/FOXK1 axis promoted lung adenocarcinoma progression. Am J Transl Res.

[4] Wang J, Zhao X, Wang Y, Ren F, Sun D, Yan Y (2020). circRNA-002178 act as a ceRNA to promote PDL1/PD1 expression in lung adenocarcinoma. Cell Death Dis.

[5] Wang HL, Wang HR, Liang Y, Hu AN, Enguita FJ, Zhou XG (2020). Hsa_circ_0006571 promotes spinal metastasis through sponging microRNA-138 to regulate sirtuin 1 expression in lung adenocarcinoma. Transl Lung Cancer Res.

[6] Liu S, Liu X, Li J, Zhou H, Carr MJ, Zhang Z (2019). Long noncoding RNAs: novel regulators of virus–host interactions. Rev Med Virol.

[7] Shields EJ, Petracovici AF, Bonasio R (2019). lncRedibly versatile: biochemical and biological functions of long noncoding RNAs. Biochem J.

[8] Guan H, Zhu T, Wu S, Liu S, Liu B, Wu J (2019). Long noncoding RNA LINC00673-v4 promotes aggressiveness of lung adenocarcinoma via activating WNT/β-catenin signaling. Proc Natl Acad Sci U S A.

[9] Deng X, Xiong W, Jiang X, Zhang S, Li Z, Zhou Y (2020). LncRNA LINC00472 regulates cell stiffness and inhibits the migration and invasion of lung adenocarcinoma by binding to YBX1. Cell Death Dis.

[10] Wang D, Liu K, Chen E (2020). LINC00511 promotes proliferation and invasion by sponging miR-515-5p in gastric cancer. Cell Mol Biol Lett.

[11] Lu G, Li Y, Ma Y, Lu J, Chen Y, Jiang Q (2018). Long noncoding RNA LINC00511 contributes to breast cancer tumourigenesis and stemness by inducing the miR-185-3p/E2F1/Nanog axis. J Exp Clin Cancer Res.

[12] Zhu FY, Zhang SR, Wang LH, Wu WD, Zhao H (2019). LINC00511 promotes the progression of non-small cell lung cancer through downregulating LATS2 and KLF2 by binding to EZH2 and LSD1. Eur Rev Med Pharmacol Sci.

[13] Xue J, Zhang F (2020). LncRNA LINC00511 plays an oncogenic role in lung adenocarcinoma by regulating PKM2 expression via sponging miR-625-5p. Thorac Cancer.

[14] Salmena L, Poliseno L, Tay Y, Kats L, Pandolfi PP (2011). A ceRNA hypothesis: the Rosetta Stone of a hidden RNA. language? Cell.

[15] Zhao W, Jiang X, Yang S (2020). lncRNA TUG1 promotes cell proliferation, migration, and invasion in hepatocellular carcinoma via regulating miR-29c-3p/COL1A1 axis. Cancer Manag Res.

[16] Siriwardhana C, Khadka VS, Chen JJ, Deng Y (2019). Development of a miRNA-seq based prognostic signature in lung adenocarcinoma. BMC Cancer.

[17] Chen Q, Chen S, Zhao J, Zhou Y, Xu L (2021). MicroRNA-126: a new and promising player in lung cancer. Oncol Lett.

[18] Yang R, Zhou Y, Du C, Wu Y (2020). Bioinformatics analysis of differentially expressed genes in tumor and paracancerous tissues of patients with lung adenocarcinoma. J Thorac Dis.

[19] Sun J, Liu J, Zhu Q, Xu F, Kang L, Shi X (2020). Hsa_circ_0001806 Acts as a ceRNA to facilitate the stemness of colorectal cancer cells by increasing COL1A1. Onco Targets Ther.

[20] Liu T, Ye P, Ye Y, Lu S, Han B (2020). Circular RNA hsa_circRNA_002178 silencing retards breast cancer progression via microRNA-328-3p-mediated inhibition of COL1A1. J Cell Mol Med.

[21] Dong S, Zhu P, Zhang S (2020). Expression of collagen type 1 alpha 1 indicates lymph node metastasis and poor outcomes in squamous cell carcinomas of the lung. PeerJ.

[22] Du X, Tu Y, Liu S, Zhao P, Bao Z, Li C (2020). LINC00511 contributes to glioblastoma tumorigenesis and epithelial-mesenchymal transition via LINC00511/miR-524-5p/YB1/ZEB1 positive feedback loop. J Cell Mol Med.

[23] Zhao X, Liu Y, Li Z, Zheng S, Wang Z, Li W (2018). Linc00511 acts as a competing endogenous RNA to regulate VEGFA expression through sponging hsa-miR-29b-3p in pancreatic ductal adenocarcinoma. J Cell Mol Med.

[24] Yan L, Wu X, Liu Y, Xian W (2018). LncRNA Linc00511 promotes osteosarcoma cell proliferation and migration through sponging miR-765. J Cell Biochem.

[25] Kou J, Zheng X, Guo J, Liu Y, Liu X (2020). MicroRNA-218-5p relieves postmenopausal osteoporosis through promoting the osteoblast differentiation of bone marrow mesenchymal stem cells. J Cell Biochem.

[26] Huang JL, Cao SW, Ou QS, Yang B, Zheng SH, Tang J (2018). The long non-coding RNA PTTG3P promotes cell growth and metastasis via up-regulating PTTG1 and activating PI3K/AKT signaling in hepatocellular carcinoma. Mol Cancer.

[27] Zhang C, Liu S, Wang X, Liu H, Zhou X, Liu H (2021). COL1A1 is a potential prognostic biomarker and correlated with Immune infiltration in mesothelioma. Biomed Res Int.

[28] Liu H, Wang S, Zhou S, Meng Q, Ma X, Song X (2019). Drug resistance-related competing interactions of lncRNA and mRNA across 19 cancer types. Mol Ther Nucleic Acids.

[29] Qiu HB, Yang K, Yu HY, Liu M (2019). Downregulation of long non-coding RNA XIST inhibits cell proliferation, migration, invasion and EMT by regulating miR-212-3p/CBLL1 axis in non-small cell lung cancer cells. Eur Rev Med Pharmacol Sci.

[30] Sun CB, Wang HY, Han XQ, Liu YN, Wang MC, Zhang HX (2020). LINC00511 promotes gastric cancer cell growth by acting as a ceRNA. World J Gastrointest Oncol.

[31] Zhang X, Wang Y, Zhao A, Kong F, Jiang L, Wang J (2020). Long non-coding RNA LINC00511 accelerates proliferation and invasion in cervical cancer through targeting miR-324-5p/DRAM1 axis. Onco Targets Ther.

[32] Yang Y, Ding L, Hu Q, Xia J, Sun J, Wang X (2017). MicroRNA-218 functions as a tumor suppressor in lung cancer by targeting IL-6/STAT3 and negatively correlates with poor prognosis. Mol Cancer.

[33] Peng Z, Pan L, Niu Z, Li W, Dang X, Wan L (2017). Identification of microRNAs as potential biomarkers for lung adenocarcinoma using integrating genomics analysis. Oncotarget.

[34] Li Y, Shi B, Dong F, Zhu X, Liu B, Liu Y (2021). LncRNA KCNQ1OT1 facilitates the progression of bladder cancer by targeting MiR-218-5p/HS3ST3B1. Cancer Gene Ther.

[35] Zare A, Ahadi A, Larki P, Omrani MD, Zali MR, Alamdari NM (2018). The clinical significance of miR-335, miR-124, miR-218 and miR-484 downregulation in gastric cancer. Mol Biol Rep.

[36] Jiang K, Liu H, Xie D, Xiao Q (2019). Differentially expressed genes ASPN, COL1A1, FN1, VCAN and MUC5AC are potential prognostic biomarkers for gastric cancer. Oncol Lett.

[37] Ma HP, Chang HL, Bamodu OA, Yadav VK, Huang TY, Wu ATH (2019). Collagen 1A1 (COL1A1) is a reliable biomarker and putative therapeutic target for hepatocellular carcinogenesis and metastasis. Cancers (Basel).

[38] Oleksiewicz U, Liloglou T, Tasopoulou KM, Daskoulidou N, Gosney JR, Field JK (2017). COL1A1, PRPF40A, and UCP2 correlate with hypoxia markers in non-small cell lung cancer. J Cancer Res Clin Oncol.

[39] Geng Q, Shen Z, Li L, Zhao J (2021). COL1A1 is a prognostic biomarker and correlated with immune infiltrates in lung cancer. PeerJ.

[40] Zhong ME, Chen Y, Zhang G, Xu L, Ge W, Wu B (2019). LncRNA H19 regulates PI3K-Akt signal pathway by functioning as a ceRNA and predicts poor prognosis in colorectal cancer: integrative analysis of dysregulated ncRNA-associated ceRNA network. Cancer Cell Int.

[41] Wang Q, Mao X, Luo F, Wang J (2021). LINC00511 promotes gastric cancer progression by regulating SOX4 and epigenetically repressing PTEN to activate PI3K/AKT pathway. J Cell Mol Med.

