# Supplementary material for: LncRNA LINC00511 promotes COL1A1-mediated proliferation and metastasis by sponging miR-126-5p/miR-218-5p in lung adenocarcinoma
Source: BMC Pulm Med. 2022 Jul 16;22:272. doi: 10.1186/s12890-022-02070-3 (PMC9287882; doi:10.1186/s12890-022-02070-3)
Supplement: Supplementary file 1 — Additional file 1. Original results of western blot assays in the laboratory. [file 12890_2022_2070_MOESM1_ESM.docx]

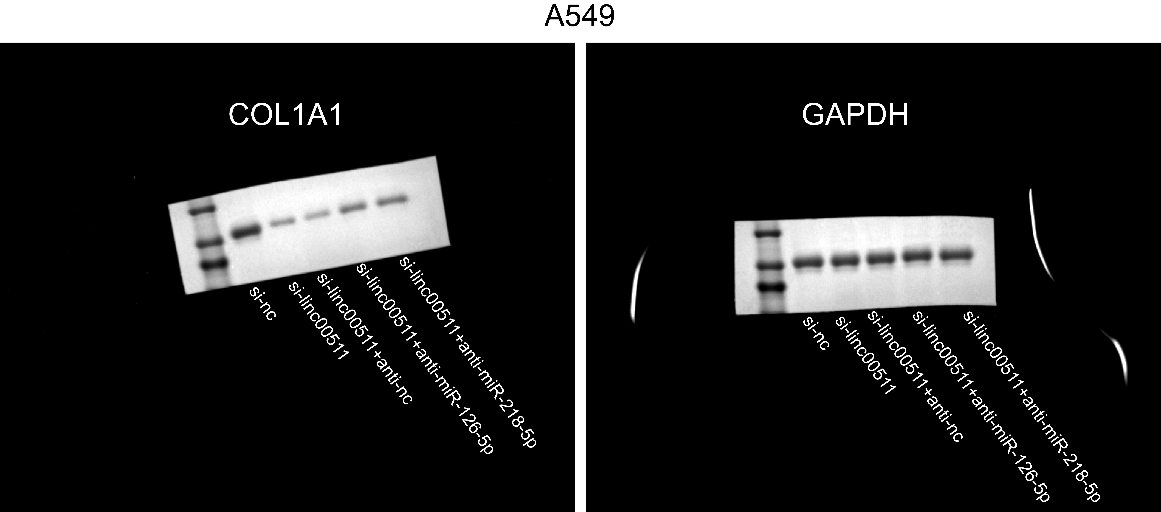


Uncropped and unedited versions of the blots in Figure 5.


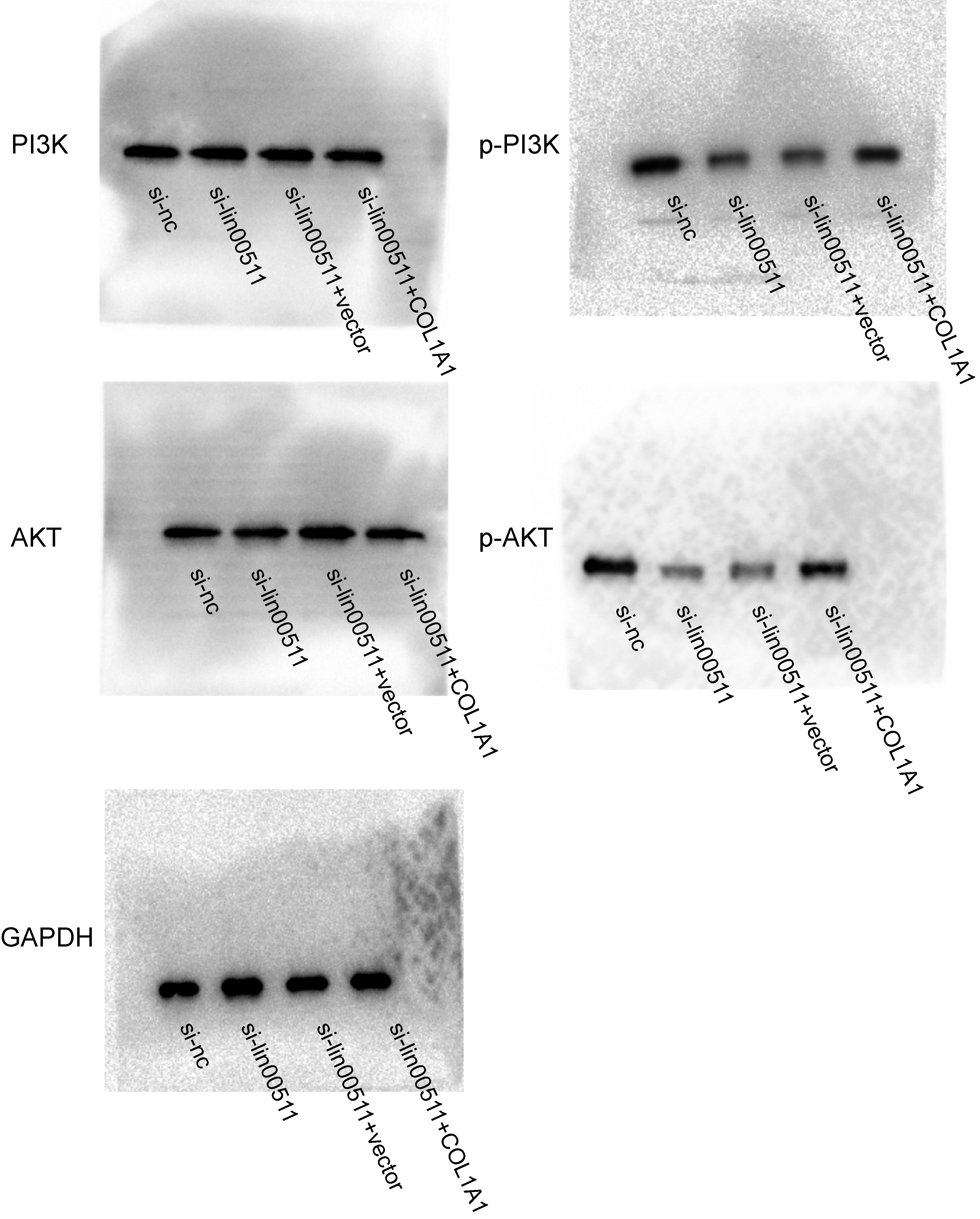


Uncropped and unedited versions of the blots in Figure 6.
